# Ultrasound in Prenatal Diagnostics and Its Impact on the Epidemiology of Spina Bifida in a National Cohort from Denmark with a Comparison to Sweden

**DOI:** 10.1155/2018/9203985

**Published:** 2018-02-01

**Authors:** Charlotte Rosenkrantz Bodin, Mikkel Mylius Rasmussen, Ann Tabor, Lena Westbom, Eleonor Tiblad, Charlotte Kvist Ekelund, Camilla Bernt Wulff, Ida Vogel, Olav Bjørn Petersen

**Affiliations:** ^1^Department of Gynecology and Obstetrics, Aarhus University Hospital, Aarhus, Denmark; ^2^Department of Neurosurgery, Aarhus University Hospital, Aarhus, Denmark; ^3^Center of Fetal Medicine, Department of Obstetrics, Copenhagen University Hospital, Rigshospitalet, Copenhagen, Denmark; ^4^Section of Pediatrics, Department of Clinical Sciences Lund, Faculty of Medicine, Skåne University Hospital, Lund University, Lund, Sweden; ^5^Department of Fetal Medicine, Karolinska University Hospital, Stockholm, Sweden; ^6^Department of Clinical Genetics, Aarhus University Hospital, Aarhus, Denmark; ^7^Center for Prenatal Diagnostics, Aarhus University Hospital, Aarhus, Denmark

## Abstract

**Objectives:**

The aim of this study was to assess the incidence, the prenatal detection rate by ultrasound, and the pregnancy outcome of spina bifida (SB) in Denmark (DK) in 2008–2015 and to compare results to national data from Sweden.

**Methods:**

Data were retrieved from the Danish Fetal Medicine Database, which includes International Classification of Diseases- (ICD-) 10 codes for pre- or postnatally diagnoses and pregnancy outcome. Missing data were obtained from the National Patient Register. Livebirth data with myelomeningocele (MMC) in Sweden were obtained from different databases.

**Results:**

There were 234 cases with SB in DK in 2008–2015. The incidence of SB was 4.9 : 10,000; 89% were detected with ultrasound prior to week 22; 90% of these pregnancies were terminated (ToP); 91% were isolated malformations of which 11% showed abnormal karyotype. The incidence of newborns with MMC was 1.3 : 10,000 in Sweden.

**Conclusions:**

Ultrasound screening has a major impact on the epidemiology of SB. The prenatal detection rate of SB was high, and most SB cases were isolated and had a normal karyotype. Among women with a prenatal fetal diagnosis of SB, 90% chose to have ToP. The incidence of newborns with SB was higher in Sweden than in DK.

## 1. Introduction

Since the introduction of new guidelines for prenatal diagnostics in 2004, all pregnant women in Denmark have been offered a prenatal screening program. The program comprises two ultrasound scans during their pregnancy: one in gestational weeks 11–14 (scanning primarily for chromosomal abnormalities) and one in gestational weeks 18–21 (scanning primarily for malformations) as well as midwife consultations between the scans [1]. These guidelines were issued by the Danish Health Authority in 2004. They were not designed to eradicate disease but to support pregnant women's reproductive autonomy, confirm normality by offering ultrasound and possibly genetic testing, facilitate planning of optimal postnatal care, and give women the possibility of applying for late termination of pregnancy (ToP) in case of severe fetal disease, like spina bifida (SB), within week 22 + 0. The current screening uptake is approximately 97%.

SB is a birth defect in the group of neural tube defects (NTD). It results from failed closure of the neural folds during the first month of gestation. SB is associated with severe morbidity and mortality, depending on the type, size, and site of the lesion [2, 3]. The incidence of SB differs globally from 1.7 to 19 : 10,000 fetuses [4–12]. SB can be detected in the second trimester using ultrasound which will reveal specific cranial signs of the cerebellum and the skull [13]. Previous studies have shown that intake of folic acid during pregnancy may decrease the risk of SB [6, 7]. This has made some countries use mandatory food fortification to ensure adequate supply of folic acid to pregnant women [6, 14]. In Denmark, folic acid is recommended as a food supplement from the day pregnancy is planned, but it is not added to food as part of a mandatory scheme. Danish folic acid recommendations have shown no impact on the incidence of SB fetuses in western parts of the country due to Danish pregnant women not complying with the guidelines and thus not getting the recommended amount of folic acid [15–17]. Even so, one of these studies showed a decrease in the incidence of infants born with SB after year 2006, that is, at the same time as the above-mentioned prenatal screening program was introduced at all obstetric departments in Denmark [16]. This suggests that the use of ultrasound scans may have an impact on the epidemiology of SB in Denmark.

Different countries have developed various prenatal ultrasound strategies to manage pregnancies complicated by SB. In Sweden, another Scandinavian country with a population approximately twice the size of the Danish population and similar legislation regarding ToP, second-trimester ultrasound scan is also offered to all pregnant women. In Sweden, however, the proportion of women who accept prenatal ultrasound screening varies more across regions than in Denmark.

The objectives of this study were to estimate the true incidence of SB in Denmark, to assess the detection rate at first-trimester and second-trimester ultrasound screening, to identify pregnancy outcomes in years 2008–2015, and to identify similarities as well as differences in incidence of SB between Denmark and Sweden.

Overlapping and sometimes inconsistent terms are used for spinal NTDs [18]. In the present study, the term “spina bifida” includes all open spinal NTDs and meningocele and lipomatous malformations with neurological deficits of the skin-covered SB.

## 2. Method

### 2.1. The Danish Fetal Medicine Database

Data for this study were primarily obtained from the Danish Fetal Medicine Database (DFMD), which is a national database that includes data from all obstetric departments in Denmark. The DFMD contains information on all pregnancies in DK with a first-trimester scan from 1 January 2008. With an uptake of more than 90%, this corresponds to 50, -60,000 pregnancies per year. The DFMD receives information regarding maternal and pregnancy characteristics and International Classification of Diseases- (ICD-) 10 codes for any prenatally diagnosed anomaly automatically from the prenatal software system (Astraia GMBH, Munich, Germany) used in all obstetric departments. Data are linked with pre- and postnatal outcomes from the Danish Cytogenetic Register, the National Patient Register (NPR), and the National Birth Register. The DFMD thus makes it possible to analyze >90% of the entire population of pregnant women with regard to screening results and outcomes [19].

### 2.2. Data Collection

**Figure 1:**
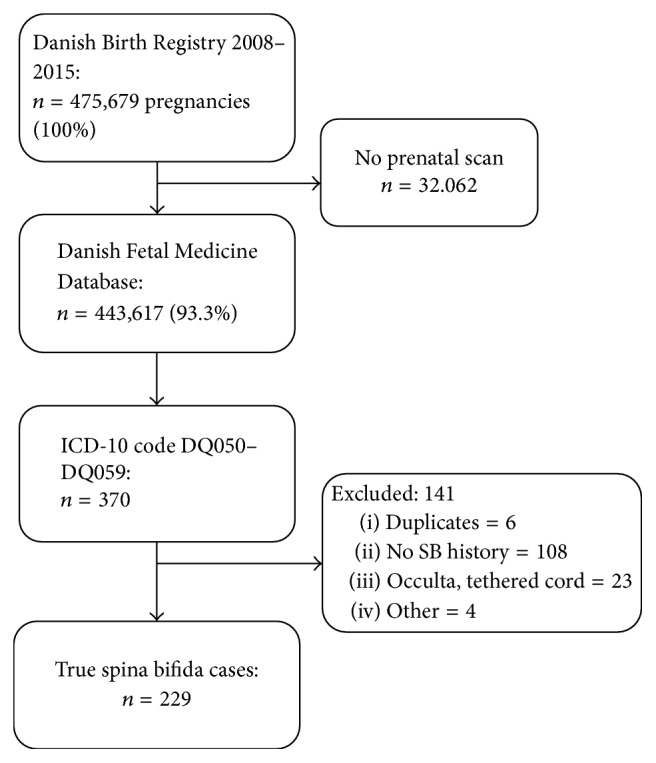
Flowchart of inclusion from the Danish Fetal Medicine Database (DFMD).

Data were collected prospectively and analyzed retrospectively. Firstly, we collected data from all pregnancies in Denmark with due date in the period from 1 January 2008 to 31 December 2015 that underwent a second-trimester ultrasound scan and had a pre- or postnatally registered International Classification of Disease- (ICD-) 10 code in the range of Q050–Q059. We performed a search of the Astraia software for cases that had ultrasound indications of SB but lacked an ICD 10-code in the searched range to identify cases that had been given an ICD 10-code not included in our search, for instance, diagnoses in the Q06 group (“other congenital malformations of the spinal cord”).  Figure 1 provides an overview of the inclusion parameters. We retrieved data regarding due date, prenatally diagnosed anomaly, anomaly diagnosed at any time postnatally until data extraction, coexistence of other malformations, karyotype, and outcome (live-born, ToP, and adverse pregnancy outcome). No information on size and site of the lesion was obtained.

**Figure 2:**
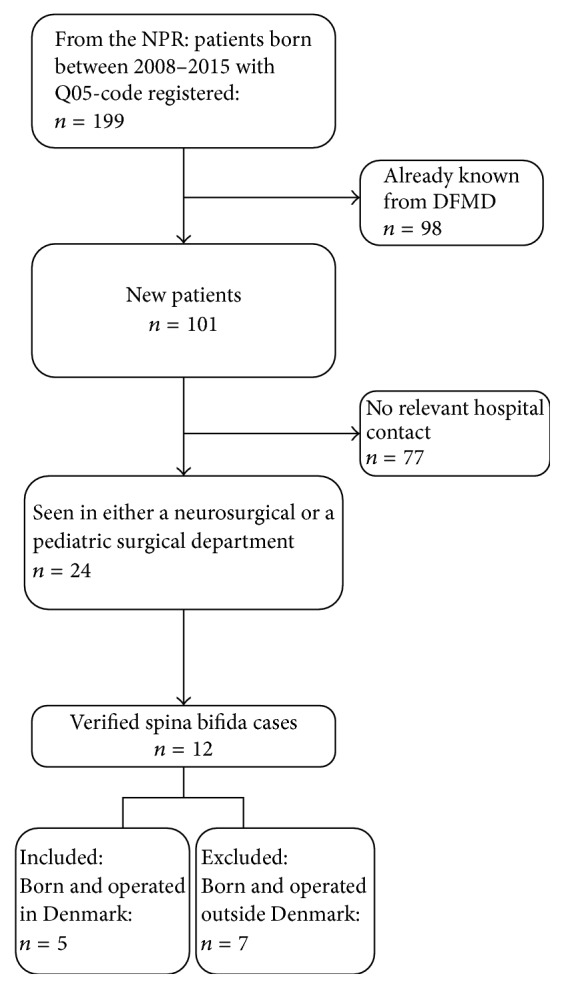
Flowchart of inclusion from the National Patient Register (NPR), DFMD: Danish Fetal Medicine Database.

Secondly, we collected all cases born in the study period with an ICD-10 code for SB from the NPR and included Civil Registration Numbers (CPR, which all citizens in Denmark are given at birth or upon immigration) not already known from the DFMD. In this way, we included SB cases among babies to the 7% of mothers who had not been scanned prenatally and therefore had not been registered in the DFMD. We also evaluated patient files for cases with a neurosurgical or pediatric surgical contact, seen in a hospital within the first year of life. See the flowchart in Figure 2.

Medical records on all infants and mothers in whom a ToP was performed (including autopsy results) were evaluated for validation. This made it possible to collect missing data, and cases were included or excluded according to the European Surveillance of Congenital Malformation (EUROCAT) guidelines [20]. This meant that we excluded all infants with SB occulta, lipomatous malformations, or tethered cord without neurological deficits, as well as all suspected but not confirmed SB cases. Babies born and having had primary surgery outside of Denmark were excluded from further statistical analysis (*n* = 7). Fetal closure of open SB had not been performed on any of these fetuses.

### 2.3. Statistical Analysis

Data were stored using RedCap software and exported to STATA® 14 for analysis. Student's *t*-test and Wilcoxon–Mann–Whitney test were used for numerical data, and chi^2^-test was used for binominal data. Variation over years was tested by a Poisson regression model, and ToP rates and detection rates were analyzed using binomial probability tests.

*p* < 0.05 was considered statistically significant; 95% confidence intervals are presented after each result in brackets.

### 2.4. Sweden

Data from Sweden were collected from the national follow-up program and quality of care registry in spinal dysraphism and hydrocephalus. The SB part of the follow-up program, called the MMCUP, includes a lifelong follow-up of body function/structures, activity, participation, treatment, and self-reported health-related quality of life for all children with SB born between 2007 and 2015 in Sweden [21]. The MMCUP provides information on live born but not unborn fetuses like the Danish registers. Hence, from Sweden, fetuses terminated by ToP or still births were not included. We extracted data from the MMCUP on SB and the prenatal diagnosis for all infants born in Sweden from 1 January 2008 to 31 December 2015.

Data on the total number of births in Sweden were obtained from the Swedish population registry, Statistics Sweden. We received extracted anonymized statistics on infants and abortions coded with Q05 from the Swedish National Board of Health and Welfare and the number of pregnancies for the years 2008–2014 from the following registers: the Medical Birth Register, the Swedish NPR, and the Surveillance Register of Birth Defects. Only patients diagnosed prenatally or within their first year of life were included. Numbers from 2015 were not available at the point of collection. Because of differences in legislation on patient anonymity between the two counties' databases, it was not possible to validate any of the Swedish cases.

### 2.5. Permissions

Permission to collect and store data was obtained from the Danish Data Protection Agency (reference number: 2012-58-006). Permission to look at the patient files of the babies of mothers who had not been scanned prenatally was granted by the Danish Patient Safety Authority (reference number: 3-3013-1721/1). Ethical approval for the MMCUP was provided in Lund, Sweden (EPN Lund, 241-2009).

## 3. Results

### 3.1. Incidence

There were 475,679 pregnancies in Denmark from 2008 to 2015. A total of 234 true SB cases were included in the study population (Figure 1). The incidence of pregnancies complicated by SB was 4.9 : 10,000 [4.3–5.6 : 10,000], and this incidence did not differ over the years 2009–2015 (*p* = 0.81), except in 2008 when there was a significantly lower incidence of pregnancies with SB (*p* = 0.03). The incidence of live-born SB cases was 0.8 : 10.000 [0.6–1.1 : 10,000] with no significant difference between the years (*p* = 0.7).

There were 7 additional cases born and treated outside Denmark but followed in a Danish hospital after immigration.

### 3.2. Prenatal Diagnosis

Of the 234 cases, 223 had an ultrasound scan done before 22 weeks, whereas 6 did not attend screening until later in pregnancy, and no information on screening was available in 5 cases. Prenatal detection at any gestational age was achieved in 93.6% [89.6–96.4%] (219/234) of cases, and 88.5% [83.7–92.5%] had been detected prior to week 22 (207/234) (stratified by years in Table 1).

We divided data on maternal characteristics from DFMD into two subgroups according to gestational age at diagnosis to rule out baseline characteristics as reason for nondetection (Table 2). Complete baseline data were available for 187 out of 207 (<week 22) and for 16 out of 22 (≥week 22), respectively. There was no significant difference between the two groups of mothers regarding age, body mass index (BMI), ethnicity, smoking status, or mode of conception. No information was obtained regarding mothers of the 5 babies with SB who had not been scanned prenatally.

The sensitivity of the Danish screening program was 92.8% [88.6–95.8%], since 207 of the 223 who attended screening were diagnosed before gestational week 22, with 15.5% [10.8–21.1%] in the first (32/207) and 84.5% (78.8–89.2%) in the second trimester (175/207). There was no statistical variation in the prenatal detection rate during the study period (*p* = 0.99).

Information about the type of SB was obtained for 14 of the 16 cases not identified at screening; in 11 of these cases (78.6%), the SB was skin-covered, meaning either meningocele or lipomatous malformations, suggesting that close to all of the opened SB cases were detected.

### 3.3. Outcome

In cases with a SB diagnosis made by ultrasound prior to week 22 of gestation, 90.3% [86.0–94.5%] (187/207) of the women opted for termination. Of all 234 cases, 190 resulted in ToP, corresponding to 81.6% [76.1–86.4%] of all SB cases in Denmark. The tendency to opt for termination did not change significantly within the study period (*p* = 0.99).

In the database, ethnicity is coded as “Caucasian” (European, Middle Eastern, North African, and Hispanic), Afro Caribbean, Asian, and Oriental. However, as there were very few numbers in the last three groups, we assembled them together in one group, calling it “Non-Caucasian.” Among mothers of Non-Caucasian origin, 30.7% chose to continue their pregnancy when SB was diagnosed before week 22. This was higher than the 6.3% in the Caucasian group (*p* = 0.0015). No difference was noted between the two groups regarding mode of conception (*p* = 0.13) or maternal age (*p* = 0.08).

A total of 20 women chose to continue their pregnancy with a SB diagnosis by ultrasound before 22 weeks. 16 of these women had a live-born baby and four miscarried. Of the 27 cases without a prenatal diagnosis before week 22, 22 had a live-born baby, one miscarried, and four opted for termination, and since the fetuses were considered not viable beyond 30 days, ToP was allowed in accordance with Danish legislation.

The resulting overall number of live births with SB was 38. Of these, 14 had a skin-covered SB. This is on average 4-5 infants with any kind of SB per year and of these 3 had open SB per year.

Of the 38 infants, 42.1% [25.7–58.6%] (16/38) had a prenatal diagnosis < week 22: that is, 57.9% did not know they were having a baby with SB.

In 91.3% [86.9–94.6%] of cases, SB was an isolated malformation (hydrocephalus and club feet are considered secondary to SB and are not counted as other malformations). A total of 119 (56.9%) of isolated cases had karyotypic or chromosomal information available, and 10.9% [5.9–18.0%] (13/119) were abnormal.

### 3.4. Results from Sweden

All live-born children with SB in Sweden from 2008 to 2015, in total 121 children, were known. Of these, 72% had an open and 28% had a skin-covered SB. A total of 905,060 babies were born in Sweden during the same period. Hence, the incidence of live-born infants with SB was 1.3 : 10,000 [1.1–1.6 : 10,000], which was higher (*p* = 0.04) than in Denmark. Information on prenatal diagnosis was available for 79% of these infants (96/121) and showed that 56.3% [46.1–66.4%] of the 96 infants had a prenatal diagnosis of SB: that, 43.7% of the mothers had no knowledge that they were expecting a baby with SB. This rate did not change over the years (*p* = 0.88).

In the databases from the National Board of Health and Welfare, which include data from 2008–2014, there were 308 unverified cases coded with SB (Q05) of whom 165 were registered as ToP. According to the MMCUP, only 97 infants born during 2008–2014 fulfilled the criteria for SB, as defined above, compared with 143 infants with a Q05 diagnosis in the national healthcare databases. Provided that all 165 ToP fetuses had SB and that all live-born children with verified SB were known (*n* = 97), the incidence of pregnancies with SB was 3.4 : 10,000 [3.0–3.8 : 10,000] during 2008–2014, and the ToP rate in Sweden based on verified SB was 63% [51.5–76.8] (165/262).

Table 3 provides an overview of the differences in results between Sweden and Denmark.

## 4. Discussion

This study is the first to cover the impact of prenatal ultrasound screening on the incidence of SB on a national level. In the Danish cohort, we found an incidence of SB of 4.9 : 10,000. The prenatal detection rate before 22 weeks was high and the majority of these women opted for ToP resulting in very few live births with SB. The rate of live births with SB was higher in Sweden than in Denmark, probably due to fewer women choosing prenatal diagnostics and possibly also a lower prenatal detection rate.

Ultrasound-detectable signs of open SB include “banana sign” of the cerebellum and “lemon sign” of the frontal skull [13]. Closed SB does not have the same impact on cranial structure as open SB and hence lacks the same ultrasound-detectable features [22]. Previous studies have shown that the sensitivity and specificity of ultrasound for open SB are close to 100% [23]. In the Danish study population, we found a sensitivity of 92.8%, and as our data also include some closed SB types, the sensitivity is expected to be lower than 100%.

In Denmark, 88.5% of the total population of SB was diagnosed before gestational week 22 and 93.9% at any gestational age. The EUROCAT society and other studies report prenatal detection rates in the range 81–90% [4, 8, 9, 24, 25]. Our overall prenatal detection rate was significantly higher than the percentage (89.3%) reported by the EUROCAT (*p* = 0.03), suggesting that the Danish prenatal screening program outperforms those of other European countries that pursue different strategies for prenatal screening for anomalies. The superior performance of the Danish program may likely be attributed to high coverage and acceptability. This is corroborated by a recent Dutch study [24] which found the same proportion of pregnant women accepting a second-trimester scan, and where 88% of SB cases are diagnosed in the second trimester.

Among all Danish SB cases, 81.6% resulted in ToP, and of those diagnosed with ultrasound before gestational week 22, 90.3% opted for termination. The ToP rate following prenatal diagnosis was in the same range as for Alsace in France (97%) [4] and the region Emilia-Romagna in Italy (92.4%) [8] (only open SB), but higher than rates reported by the EUROCAT (66%) [26], Atlanta in the US (34%) [12], and the northern parts of the Netherlands (78.6% when diagnosed in the second trimester) [24].

The Danish Spina Bifida Society (“Rygmarvsbrokforeningen af 1988”) does not have an official statement regarding ToP, which somehow underlines the liberal attitude towards ToP in Denmark and even among patients and relatives affected by SB. The different attitudes towards ToP evident between different ethnic groups in the present study and geographically may partly explain the varying global incidence of live-born SB cases.

Today, the possibility for prenatal genetic counseling is widely used for known hereditary diseases, and the introduction of prenatal ultrasound has made it possible to offer genetic counseling to parents expecting a child with a malformation, like SB, that has no known hereditary path. A chromosomal abnormality was found in 10.9% of isolated SB, which is comparable to the rates reported in similar studies [4, 8, 10]. This suggests that there is a high risk of chromosomal anomalies in these pregnancies compared with normal-appearing fetuses [27] and supports the idea that all women with a pregnancy complicated by SB should be offered chromosomal analysis and counseling from a multispecialist team.

It is important to note that previous studies included only open SB [8, 9, 24] or were inconsistent as to whether they included both open and closed SB [10–12, 28]. In our definition of SB, we included open SB and meningocele and lipomatous NTDs with neurological deficits. We did not include information on size and site of the lesion. Our study includes close to 100% of the Danish SB cases which minimizes selection bias and regional differences. The number of SB cases was lower in 2008 than in the other years, possibly due to natural variance over years or missing data in the establishment period in the beginning of 2008. All cases included in the study were validated by clinical audit and patient file review, which eliminates the risk of false-positive cases. A search of the Astraia software for cases that had ultrasound indications but lacked an ICD 10-code for SB was undertaken for the prenatally diagnosed group in an attempt to diminish the risk of underestimation due to noninclusion of false-negative cases. SB cases that died in utero earlier than the second trimester could not be included; thus, this could possibly lead to a small degree of underestimation. For the postnatally diagnosed group, there is a small risk that patients were not given a correct ICD 10-code and hence not reported to the NPR. However, since all SB patients in Denmark are referred to a university hospital, the risk of nonreporting is low. We base this argument on the observation that ICD-10 codes in Denmark serve multiple purposes, including the distribution of funding between healthcare institutions.

The Danish incidence of SB is in line with that of other western developed countries without food fortification [4, 7, 8, 24], whereas studies from other parts of the world where mandatory fortification of grain products exists show a lower total incidence of SB (USA, Canada, and Australia) [6, 12].

Validation of cases that are given ICD-codes for SB (Q05) is absolutely necessary to identify the “true” incidence of SB, as shown in Figures 1 and 2 for the Danish fetal and patient registries. As infants and abortions coded with Q05 were anonymized in the Swedish National Board of Health and Welfare data, validation of these cases could not be undertaken. However, comparison with MMCUP data regarding infants born with SB indicated that 32.2% of the Swedish Q05-coded infants did not have SB, a proportion that is about the same as that in Denmark according to the present study. About two-thirds of the infants with SB had an open, not skin-covered, defect in both countries. The comparison between Denmark and Sweden underlines the difference between using an anonymized and a nonanonymized database. The data from Sweden originate from the MMCUP, which was not anonymized and from the national populations registers, which were anonymized. Hence, 97/143 cases in the national population registers were present in the MMCUP, suggesting that 46 (32.2%) were false positive in the database. An even higher proportion of false-positives were found in the Danish Patient Registers after full validation of the nonanonymized data, with 108/229 (47.2%) having a wrong diagnosis.

Sweden has the same folic acid recommendations as Denmark [28], and the lower incidence of pregnancies with SB in Sweden than in Denmark (3.4 versus 4.9 : 10,000, *p* < 0.001, Table 3) must therefore be due to other factors. A previous Swedish study found a national incidence of SB of 5.44 : 10,000 during 1999–2002 [28], which is significantly higher than the estimated incidence based on the present 308 pregnancies with unverified SB. So, poor identification of registered pregnancies may have contributed to the low incidence figures for the years 2008–2014 in the present study. Despite the possibly lower incidence of pregnancies with SB, the higher incidence of infants born with SB in Sweden than in Denmark may be explained by a lower percentage of parents choosing prenatal diagnostics, lower detection by ultrasound, and possibly different attitudes towards ToP.

To our knowledge, valid national prevalence figures on SB cases with prenatal ultrasound findings did not exist prior to this study, either in Denmark or in any other country. The present study shows that a full national prenatal ultrasound screening with a high uptake has a major impact on the incidence of SB because of the high detection rate of SB by ultrasound and because a large proportion of women opt for ToP. This study may have implications for the organization of SB prenatal care, surgery, and treatment. Since the patient volume is low with an average of only four new patients per year, patients who need lifelong treatment may be better handled in a few dedicated centers to optimize the expertise of healthcare professionals and to ensure better quality of life for patients.

## 5. Conclusion

This study includes all fetal SB cases in Denmark during the years 2008–2015. The study shows that, by using ultrasound screening, almost all cases of a SB can be detected. In a country like Denmark where ToP is regulated by law, prenatal ultrasound screening may have an impact on the number of live births of children with ultrasound-detectable malformations, and it may inform healthcare professional and parental decisions with regard to ToP and the planning of postnatal care for the newborn. The difference between Denmark and Sweden, where acceptance of prenatal ultrasound screening is lower, underlines the effect of a nationwide screening program on the epidemiology of SB.

## Figures and Tables

**Table 1:** Spina bifida cases and detection divided in years; ^*∗*^Poisson regression.

Year	Total SB cases	Prenatal diagnosis	Prenatal diagnosis < week 22
2008	15	14 (93.3%)	13 (86.7%)
2009	32	29 (90.6%)	27 (84.4%)
2010	38	35 (92.1%)	34 (89.4%)
2011	33	30 (90.9%)	28 (84.8%)
2012	28	27 (96.4%)	26 (92.9%)
2013	24	21 (87.5%)	21 (87.5%)
2014	35	34 (97.1%)	31 (88.6%)
2015	29	29 (100%)	27 (93.1%)
Total	234	219 (93.6%)	207 (88.5%)
*p* value		0.99^*∗*^	0.99^*∗*^

**Table 2:** Maternal characteristics from the Danish Fetal Medicine Database; ^*∗*^Wilcoxon–Mann–Whitney test, ^*∗∗*^Student's *t*-test, ^*∗∗∗*^chi^2^ test; ^∧^Caucasian: European, Middle Eastern, North African, Hispanic; ^∧∧^Non-Caucasian: Afro Caribbean, Asian, Oriental.

Characteristic	<week 22	≥week 22	*p* value
	*N* = 187	*N* = 16	
Maternal age, years [95% CI]	29.7 [29.0; 30.4]	29.8 [27.4; 32.1]	0.88^*∗*^
BMI, [95% CI]	25.6 [24.7; 26.5]	28.1 [24.9; 31.3]	0.08^*∗∗*^
Smokers, % [95% CI]	3.8 [1.5; 7.6]	6.2 [0.2; 30.2]	0.62^*∗∗∗*^
Ethnicity, % [95% CI]			
(i) Caucasian^∧^	93.2 [88.6; 96.3]	86.7 [59.5; 98.3]	0.35^*∗∗∗*^
(ii) Non-Caucasian^∧∧^	6.8 [3.7; 11.4]	13.3 [1.7; 40.5]	
Conception, % [95% CI]			
(i) Spontaneous	90.1 [84.8; 94.0]	93.8 [69.8; 99.8]	0.63^*∗∗∗*^
(ii) Fertility treatment	9.9 [6.0; 15.2]	6.3 [0.2; 30.2]	

**Table 3:** Overview of differences between Sweden and Denmark 2008–2015; ^*∗*^2008–2014; ^*∗∗*^majority in third trimester; *p* value calculated by Student's *t*-test.

	Denmark	Sweden	*p* value
Incidence fetuses	4.92 : 10,000	3.4 : 10,000^*∗*^	0.0001
Incidence live births	0.8 : 10,000	1.3 : 10,000	0.04
ToP rate	81.2%	63.0%^*∗*^	0.02
Prenatal detection RATE among live births	42.1%	56.3%^*∗∗*^	0.11

## Abbreviations

BMI: Body mass index
DFMD: Danish Fetal Medicine Database
EUROCAT: European Surveillance of Congenital Malformations
GA: Gestational age
ICD: International Classification of Diseases
MMC: Myelomeningocele
NPR: National Patient Registry (“Landspatientregisteret”)
NTD: Neural tube defects
SB: Spina bifida
ToP: Termination of pregnancy.

## Additional Points

*Key Message*. In a national cohort from Denmark, >80% of fetal spina bifida cases were detected by prenatal ultrasound with 90% of parents choosing termination of pregnancy in case of open spina bifida, so very few infants with this malformation were born.

## References

[1] Ekelund C. K., Petersen O. B., Jorgensen F. S. (2015). The Danish Fetal Medicine Database: Establishment, organization and quality assessment of the first trimester screening program for trisomy 21 in Denmark 2008-2012. *Acta Obstetricia et Gynecologica Scandinavica*.

[2] Bowman R. M., McLone D. G., Grant J. A., Tomita T., Ito J. A. (2001). Spina bifida outcome: a 25-year prospective. *Pediatric Neurosurgery*.

[3] Oakeshott P., Hunt G. M., Poulton A., Reid F. (2010). Expectation of life and unexpected death in open spina bifida: A 40-year complete, non-selective, longitudinal cohort study. *Developmental Medicine & Child Neurology*.

[4] Timbolschi D., Schaefer E., Monga B. (2015). Neural tube defects: The experience of the registry of congenital malformations of Alsace, France, 1995-2009. *Fetal Diagnosis and Therapy*.

[5] Au K. S., Ashley-Koch A., Northrup H. (2010). Epidemiologic and genetic aspects of spina bifida and other neural tube defects. *Developmental Disabilities Research Reviews*.

[6] Atta C. A. M., Fiest K. M., Frolkis A. D. (2016). Global birth prevalence of spina bifida by folic acid fortification status: A systematic review and meta-analysis. *American Journal of Public Health*.

[7] Khoshnood B., Loane M., De Walle H. (2015). Long term trends in prevalence of neural tube defects in Europe: Population based study. *BMJ*.

[8] Ghi T., Cocchi G., Conti L. (2015). Prenatal diagnosis of open spina bifida in Emilia-Romagna. *Fetal Diagnosis and Therapy*.

[9] Salvador J., Arigita M., Carreras E., Lladonosa A., Borrell A. (2011). Evolution of prenatal detection of neural tube defects in the pregnant population of the city of Barcelona from 1992 to 2006. *Prenatal Diagnosis*.

[10] Domrose C. M., Bremer S., Buczek C. (2016). Termination of pregnancy after prenatal diagnosis of spina bifida: a German perspective. *Archives of Gynecology and Obstetrics*.

[11] Bhide P., Sagoo G. S., Moorthie S., Burton H., Kar A. (2013). Systematic review of birth prevalence of neural tube defects in India. *Birth Defects Research Part A: Clinical and Molecular Teratology*.

[12] Besser L. M., Williams L. J., Cragan J. D. (2007). Interpreting changes in the epidemiology of anencephaly and spina bifida following folic acid fortification of the U.S. grain supply in the setting of long-term trends, Atlanta, Georgia, 1968-2003. *Birth Defects Research Part A - Clinical and Molecular Teratology*.

[13] Nicolaides K. H., Campbell S., Gabbe S. G., Guidetti R. (1986). Ultrasound screening for spina bifida: cranial and cerebellar signs. *The Lancet*.

[14] Padmanabhan R. (2006). Etiology, pathogenesis and prevention of neural tube defects. *Congenital Anomalies*.

[15] Rasmussen M. M., Clemmensen D. (2010). Folic acid supplementation in pregnant women. *Ugeskr Laeger*.

[16] Clemmensen D., Thygesen M., Rasmussen M. M., Fenger-Grøn M., Petersen O. B., Mosdal C. (2011). Decreased incidence of myelomeningocele at birth: Effect of folic acid recommendations or prenatal diagnostics?. *Child's Nervous System*.

[17] Friberg A. K., Jorgensen F. S. (2015). Few Danish pregnant women follow guidelines on periconceptional use of folic acid. *Danish Medical Journal*.

[18] McComb J. G. (2015). A practical clinical classification of spinal neural tube defects. *Child's Nervous System*.

[19] Hyett J. A. (2015). The Danish Fetal Medicine Database: Revealing the fruits of collaborative research. *Acta Obstetricia et Gynecologica Scandinavica*.

[20] EUROCAT. Detailed Congenital Anomaly Coding Guidelines 2013 [cited 2017 December 16], http://www.eurocat-network.eu/content/EUROCAT-Guide-1.4-Section-3.5.pdf

[21] Alriksson-Schmidt A. I., Arner M., Westbom L. (2017). A combined surveillance program and quality register improves management of childhood disability. *Disability and Rehabilitation*.

[22] Ghi T., Pilu G., Falco P. (2006). Prenatal diagnosis of open and closed spina bifida. *Ultrasound in Obstetrics & Gynecology*.

[23] Lennon C. A., Gray D. L. (1999). Sensitivity and specificity of ultrasound for the detection of neural tube and ventral wall defects in a high-risk population. *Obstetrics & Gynecology*.

[24] Fleurke-Rozema J. H., Vogel T. A., Voskamp B. J. (2014). Impact of introduction of mid-trimester scan on pregnancy outcome of open spina bifida in the Netherlands. *Ultrasound in Obstetrics & Gynecology*.

[25] EUROCAT. Prenatal Detection Rates 2017 [cited 2017 2 December], http://www.eurocat-network.eu/prenatalscreeninganddiagnosis/prenataldetection(pd)rates

[26] EUROCAT. Prevalence Tables 2015 [cited 2017 2 December], http://www.eurocat-network.eu/accessprevalencedata/prevalencetables

[27] Ferreira J. C. P., Grati F. R., Bajaj K. (2016). Frequency of fetal karyotype abnormalities in women undergoing invasive testing in the absence of ultrasound and other high-risk indications. *Prenatal Diagnosis*.

[28] Amini H., Axelsson O., Ollars B., Anneren G. (2009). The Swedish Birth Defects Registry: Ascertainment and incidence of spina bifida and cleft lip/palate. *Acta Obstetricia et Gynecologica Scandinavica*.

